# Health and medical research funding agencies’ promotion of public engagement within research: a qualitative interview study exploring the United Kingdom context

**DOI:** 10.1186/s12961-016-0093-4

**Published:** 2016-03-24

**Authors:** Jennifer E. van Bekkum, Gillian M. Fergie, Shona Hilton

**Affiliations:** MRC/CSO Social and Public Health Sciences Unit, University of Glasgow, 200 Renfield Street, Glasgow, G2 3QB UK

**Keywords:** Funding, Health, Policy, Public engagement, Qualitative research, Research

## Abstract

**Background:**

Public engagement (PE) has become a common feature of many liberal governmental agendas worldwide. Since the turn of this century there has been a succession of United Kingdom policy initiatives to encourage research funding agencies, universities and researchers to reconsider how they engage with citizens and communities. Although most funding agencies now explicitly promote PE within research, little empirical work has been carried out in this area. In this study, we explored why and how health and medical research funding agencies in the United Kingdom have interpreted and implemented their role to promote PE within research.

**Methods:**

Semi-structured interviews were carried out with 30 key informants from 10 agencies that fund health or medical research. Data were also gathered from agencies’ websites and documentation. The analysis was based on the constant comparative method.

**Results:**

Across agencies, we found that PE was being interpreted and operationalised in various different ways. The terminology used within funding agencies to describe PE seems to be flexibly applied. Disciplinary differences were evident both in the terminology used to describe PE and the drivers for PE highlighted by participants – with applied health science funders more aligned with participatory models of PE. Within the grant funding process PE was rarely systematically treated as a key component of research. In particular, PE was not routinely incorporated into the planning of funding calls. PE was more likely to be considered in the application and assessment phases, where it was largely appraised as a tool for enhancing science. Concerns were expressed regarding how to monitor and evaluate PE within research.

**Conclusions:**

This study suggests funding agencies working within specific areas of health and medicine can promote particular definitions of PE and aligned practices which determine the boundaries in which researchers working in these areas understand and practice PE. Our study also highlights how the research grant process works to privilege particular conceptions of PE and its purpose. Tensions are evident between some funders’ core concepts of traditional science and PE, and they face challenges as they try to embed PE into long-standing systems that prioritise particular conceptions of ‘scientific excellence’ in research.

## Background

For the potential of health and medical research to be realised, it is increasingly acknowledged that scientists should engage with patients and the public. In particular, by inviting the public (sometimes described as patients, consumers or users) to provide input, researchers are afforded an opportunity to increase the timeliness and relevance of their research and generate insights for policy and practice that are implementable [1, 2]. Furthermore, engagement with groups of individuals with an interest in the subject of research has been cited as contributing to improving the processes of research design, participant recruitment, and communication and dissemination [3]. Accordingly, public engagement (PE) in healthcare, health policy and research has become a common feature of many liberal governmental agendas worldwide [4, 5].

Commonly, PE is defined as “*a two-way process, involving interaction and listening, with the goal of generating mutual benefit*” [6]. However, in practice, the term is sometimes used more loosely to describe a broad range of public-facing activities with differing levels of interactivity [7]. For example, Trench has characterised three dominant models in science communication that can be associated with broader conceptions of PE, which he terms deficit, dialogue and participation [8]. Deficit refers to one-way dissemination of information that assumes public ignorance and dissent towards science and a lack of scientific understanding and education. Dialogue refers to two-way talking and listening that assumes scientists should find out public views. Finally, participation refers to two-way deliberation that assumes that scientists and public should jointly shape issues, set agendas and negotiate meanings.

Although there has been a positive shift in the theoretical debates and advancements in thinking about public PE within science [9–12], in practice, the deficit model approach still seems to remain most widespread [11, 13–17]. While this model has been branded as inadequate and outmoded [15], it has been suggested that there is some public demand for ‘one-way’ information and that science literacy in itself can empower individuals to make more informed health decisions [8].

Within the United Kingdom context, PE has been promoted in healthcare policy since the founding of the Community Health Councils in the 1970s [18]. The PE agenda became more pervasive within the broader United Kingdom political climate in the 1990s with New Labour’s framing of citizen participation as a tool for democratic renewal and modernisation to rejuvenate relationships between state and society, and increase public accountability and legitimacy [19].

The term PE was popularised in the science policy context by the internationally influential United Kingdom House of Lords Third Report, which was published in 2000 and recommended cultivating two-way dialogue between science and the public [15]. The report advocated that PE should become an integral part of science-based policymaking and research practices. This shift in rhetoric towards active dialogue recognised the potential of bringing constructive and socially-reflexive practices of citizenship and knowledge production into mainstream science culture [12]. As a result, over the last decade, PE has become an increasingly salient feature of contemporary scientific research practice [11]. Previous research has explored a range of perspectives on PE, including drivers for PE, opportunities for PE and the impact of PE on research processes and outcomes. Delgado et al.’s [14] review of tensions between the theoretical underpinnings of PE and PE in practice highlights the most common, and potentially competing, rationales for PE: “*to achieve a predefined end; to produce a better result; and because it is the ‘right thing to do’*” (p. 831). The degree and methods of involvement in research have also been examined [20, 21]. Within the healthcare context, studies have explored how guidelines, research design, patient information and policy decision-making can be influenced by patient or public involvement [20]. Similarly, a recent review of the impact of engaging patients in health and social care research reported a range of positive impacts throughout the course of research, but suggested a key challenge was in harmonising patients’ views on the research agenda with scientific objectives [21]. Despite this research, there continues to be limitations to the evidence regarding the value of PE to both science and society, which stems from an inconsistent reporting [21], and a lack of well-developed indicators and rigorous evaluation [22, 23].

In the United Kingdom, non-commercial health and medical research funding is managed and distributed by various organisations, including governmental agencies such as Research Councils, academic associations such as learned societies, and medical research charities. Holding powers to shape research practices via mechanisms such as their control and governance of research grants, funding agencies are influential institutions within the science domain [24, 25]. In line with national policy recommendations, most funding agencies explicitly endorse and promote PE within research [26].

Since the turn of this century there has been a succession of policy-driven directives and initiatives in the United Kingdom to encourage research funders and universities to make science more socially embedded and reconsider how they engage with and relate to wider communities [27–30]. For example, the United Kingdom research funding sector has led the way internationally with initiatives such as the ‘impact agenda’ (which formalised PE within the Research Excellence Framework as an accountability measure for university funding) [29] and the Concordat for Engaging the Public with Research (which was created to demonstrate funders’ PE commitments and expectations to universities and researchers) [30]. Indeed, how PE has developed and continues to do so within the United Kingdom research funding sector is of international significance.

To date, little research has investigated research funding agencies’ own PE policies and practices. The limited studies that have been carried out within the broader sphere of science communication indicate that, in the United Kingdom and internationally, a gap exists between funding agencies’ science communication policies and the operationalisation of these in practice [2, 26, 31, 32]. For example, funders’ science communication and PE policies were not found to be systematically applied and monitored within the research grant process [26, 32] – a key interface in which funders can directly influence and shape research practices. Further, there was little researcher accountability for the execution and quality of their PE plans within this process [32, 33]. A more recent study found that, while PE featured as a central pillar of some United Kingdom research councils’ policies, two-way dialogue practices were not always seriously embraced by national and international research funding agencies [31]. Such findings are perhaps not surprising as some authors indicate that work to embed PE within non-commercial research funding agencies’ research structures and practices is in its infancy compared to commercial research [34, 35].

In order to address the paucity of empirical research in this area we designed a study that aimed to explore how agencies that fund health and medical research in the United Kingdom have interpreted and implemented their role to promote PE within research. By focusing on the research grant allocation process, we aimed to understand the key mechanisms by which funding agencies can directly influence and shape research practices. Specifically, we were interested in analysing:

- How health research funding agencies define PE?
- What health research funding agencies motives are for advocating PE?
- How PE features in the different phases of the research grant funding process?

## Methods

A qualitative approach was adopted and semi-structured interviews were employed to gain in-depth insights from key informants working for, or affiliated to, health or medical research funding agencies across the United Kingdom. In addition, the study drew on supplementary information from publicly accessible documents published by funding agencies and their websites.

The research project was designed with an emphasis on ‘real world’ learning. Informed by Kvale’s adoption of the concept of the ‘bricoleur’ in relation to the researcher [36], we selected elements from broadly constructivist analytical approaches that fit with the purpose of the study to explore definitions and constructions of concepts and processes [37–39]. This perspective “[assumes] *the relativism of multiple social realities, recognises the mutual creation of knowledge by the viewer and viewed, and aims toward an interpretive understanding of subjects’ meanings*” ([40], p.250).

In order to capture complexities and differences in the data we adopted practices such as constant comparative analysis, descriminant case identification and the use of field notes and memoing in our analysis [37–39]. The study was also informed by a critical axiology inherent in the underlying participatory nature of the topic, PE.

An advisory group made up of PE experts from the academic, research funding and advocacy sectors was instrumental in shaping all stages of the project. Ethical approval for the study was granted by the University of Glasgow College of Social Sciences Research Ethics Committee.

### Sample and recruitment

Using purposive sampling, we applied the following criteria for selecting funding agencies. They were required to be United Kingdom-based, non-commercial, either partially or exclusively funding health or medical research, and explicitly promoting PE within research. Funders that specifically promoted PE were of particular interest for a number of reasons. To our knowledge, the majority of non-commercial funding agencies now advocate PE; in line with some of the project’s core research questions, we wanted to learn about how PE featured in internal processes, therefore, funders who did not advocate PE would be less likely to provide information in this respect. Finally, after discussions with our advisory group it was anticipated that any funder who did not overtly advocate PE would be highly unlikely to want to take part in our study. To enable greater generalisability of findings, we aimed to build diversity into the sample by including differing sub-disciplines of health and medical research such as biomedicine, clinical studies, public and social health sciences, and health services research.

The United Kingdom Association of Medical Research Charities has 138 member charities, spending £1.3bn a year on research. There are seven United Kingdom Research Councils, of which three routinely fund health-related research, with the others often funding health and wellbeing-related research as part of cross-council initiatives. Most United Kingdom Government departments also support research, and there are a range of learned societies and academic associations in the United Kingdom that also fund health research. With the help of the advisory group, we identified 13 non-commercial funding agencies that represented a range of these research funding sources. Ten funding agencies agreed to take part, providing a core sample that comprises some major medical and health research funders, including publicly funded organisations, learned societies and medical charities. Due to the distinct profile of some funding agencies participating, the names and specific types of each agency involved in the research have been anonymised to protect the identity of the participants and their associated funding agencies. The majority of agencies in our sample, while operating autonomously, receive significant amounts of their funding from central government and work closely with national and international science/health policies and initiatives. Ensuring anonymity was a critical factor in gaining access to this group of key informants; however, we recognise that by providing anonymity for participating agencies we limit the extent to which explicit comparisons between different types of agencies can be made, and the extent to which we can contextualise our findings.

From each organisation we aimed to recruit three key informants: an individual working in a PE role, an individual working in a research funding role and a grant review committee member. By recruiting a range of participants from each funding agency, the study aimed to maximise the perspectives explored and develop a holistic understanding of the nature of PE as conceptualised and operationalised within the organisation. We recruited a total of 30 participants. The majority of participants (n = 27) worked for agencies (n = 8) that managed and awarded grants to projects. Only a minority of participants (n = 3) and agencies (n = 2) operated under different funding models.

### Data collection

Interviews were carried out between October 2012 and April 2013. Informed consent was gained from each participant prior to taking part in the study. Each interview lasted approximately 60 minutes. Most interviews were carried out face-to-face (n = 21) at participants’ workplaces or at nearby convenient locations, and a minority of interviews were carried out over the telephone (n = 5). At the request of a small number of participants (n = 4), some interviews were jointly conducted with two members of the same agency.

The interview guide was developed and tailored for each of the three different sub-groups of participants and to individual funding agencies, ensuring that questions were relevant to their professional knowledge and experience. The development of the interview guides was informed by the publicly available information provided by organisations, which provided points of departure for in-depth conversations about relevant internal workings and practical experiences. Questions were handled flexibly to allow the interviewer to explore novel areas of interest that arose during the interviews. Typically, questions covered conceptions and uses of PE; incentives and support for PE; policies, strategies and practices; influences on PE policy and practice; the research grant process; and challenges and successes.

In addition to interviews, participating agencies’ websites were systematically searched for PE relevant information, including published definitions, pledges and activities. Where available, we also accessed supplementary online documents such as organisational PE visions/strategies and grant application forms. Information gained from documents and websites was included in the analysis. Throughout the data collection and analysis phase, comprehensive field notes and memos were made.

### Data analysis

All interviews were recorded and transcribed verbatim and participants’ and agencies’ names were removed to ensure anonymity. The analysis was based on the constant comparative method and the use of discriminant cases stemming from grounded theory [40, 41]. Open codes were generated from transcripts, field notes and some relevant information from documents and websites, which were entered into a coding matrix to enable further identification of patterns both within and across accounts [39].

By including three key informants with distinct job roles from each funding agency, we were afforded a particularly rich and varied set of data, which helped to contextualise accounts [42]. For example, participants working in a PE role tended to talk in a more reflective and positive manner about how PE was being interpreted and operationalised within their agency, often discussing exemplary and flagship practices that they hailed as successes. Those working in research funding-related roles or sitting on grant review committees tended to discuss some of the more procedural and practical challenges and tensions in trying to embed PE within their research communities. Additionally, using numerous types of data enabled us to capture and respect multiple perspectives [42], and in turn to take into account complexities [38]. As discussed above, publicly available documents and website information provided contextual background information for developing the interview questions and aided the analysis. Field notes and memoing were used to aid the initial analysis, and to provide further insight around issues and discrepancies highlighted within the interviews themselves [38].

Once the coding matrix was complete, to further enhance the quality and trustworthiness of the data, a second researcher read over the codes and a sample of the transcripts with a view to asking critical questions and suggesting alternative explanations to enhance the primary researchers’ reflexivity towards the data [43]. A summary of the findings was also presented to members of the advisory group. Questions and feedback from the group helped to further enhance researchers’ reflexivity by challenging how data were grouped and how theme headings were developed [43]. Finally, to enhance the collaborative participation, fairness and transparency of the study [42], the amended findings were circulated to all interviewees, inviting them to comment via email or telephone. Five of the interviewees responded, highlighting their interest in specific points and asking for more clarity on themes or issues. This process also helped to enhance researchers’ reflexivity and resulted in some refinements to the presentation of the data.

## Results

The main findings are presented in two sections below. The first section – ‘the scope of PE’ – highlights the funders’ definitions of PE, the terminology they used to describe PE, the drivers for PE they identified, and the distinctiveness of PE strategies. The second section – ‘PE within the research grant process’ – highlights how PE is integrated into the planning, application, assessment and monitoring phases of the funding process. Quotations from participants are presented throughout to exemplify particular perspectives and ground themes and ideas raised in the analysis in participants’ accounts.

### Section one: the scope of PE

#### Definitions

Most funding agencies seemed to lack a formal definition for PE, and participants appeared to find it challenging to pin down a specific description. One participant, who held a PE role within their agency, stated: “*Yeah, there’s a reason that we don’t sort of define it on our website. And that’s because we don’t think that there is a sort of one-size-fits-all definition*”. The term PE seemed to be viewed as encompassing a wide spectrum of activities involving a variety of public and patient groups. A point of contention was how funding agencies chose to interpret the word ‘engagement’. Although dialogue was commonly acknowledged as one aspect of engagement, information provision, public relations or promotional activities were also recognised by some funding agencies as PE. For example, one participant, who held a PE role within their agency, stated:

“*There’s all the PR stuff, and communications, information and marketing. And some people say that’s not engagement. I disagree.... An awful lot of people talk that public engagement equals dialogue. Disagree with that as well.* […] *I’ve had many good arguments with my colleagues – that this public engagement is ‘two-way’* […] *and if I have to be sat in the same room with someone, to be engaged, and have to be able to question them. And I’ve watched many, many* […TV] *programmes in which I’ve been personally engaged, and it’s changed the way I’ve felt. Or I’ve been engaged because I’ve learnt something, or I’ve really connected with the presenter. I don’t need to have them there in front of me, and me give them the Spanish Inquisition about what they’re doing, in order to feel engaged*”.

This broad view, endorsing a more didactic approach to PE, was most commonly conveyed by agencies that funded significant amounts of health and medical research associated with basic and physical sciences. In contrast, for the agency that funded the most applied health research, PE was described as a participatory activity. These different framings perhaps reflect the different disciplinary norms around PE, rooted in contemporary understandings and practices of research and publics developed within the specific research community.

The unfixed nature of PE appeared to enable funding agencies to legitimately interpret and define it in ways that fitted their organisational cultures and contexts. For example, one participant who held a PE role within an agency that funds a range of medical research stated:

“*We have also started referring to our media work as public engagement work. Now, it’s not sort of – you know, strictly speaking it’s not engagement, in the sense that, you know, there’s no opportunity really for the public to ask us, you know, to ask us questions back. It’s one way, it’s really information provision. But what I want – the reason I’ve started couching it in those terms is because a lot of the press work* […] *might inform some of the decisions that they* [people] *make in their own lives*”.

Funding agencies, therefore, are playing an active role in shaping the boundaries of PE within their research communities. Through developing working definitions, which in turn influence researchers, the particular perspective of the funder on what constitutes PE can both create and stifle opportunities for PE.

#### Terminology

Drawing on interview accounts, public documents and websites, we identified 18 differing terms that referred to PE (Table 1), which were used interchangeably. Many of these terms seemed ambiguous, and very few participants provided any distinct definitions for these differing terms. One participant, who held a PE role within their agency, said: “*I think it’s quite difficult because public engagement, communications, knowledge exchange are all on a continuum. And so where one ends and where one begins, it – it’s a perception challenge*”.

**Table 1:** Terms used to refer to public engagement

Citizen engagement	Outreach
Citizen involvement	Patient involvement
Communication	Patient and public involvement
Co-creation	Public engagement
Co-production	Public involvement
Dissemination	Public participation
Impact	Public understanding
Knowledge exchange	Science and society
Knowledge translation	Science in society

In some cases, the terminology used seemed to be aligned with a particular discipline. For example, funding agencies that were oriented towards social sciences seemed more likely to speak of ‘co-production’ and ‘co-creation’, agencies oriented towards applied health research more commonly used terms like ‘patient involvement’ and ‘public involvement’. Funding agencies that were more closely aligned with basic and physical sciences sometimes used the term ‘outreach’ synonymously with PE. It would appear that the different terms were related to context-specific opportunities that different disciplines were most commonly presented with to engage with the public.

#### Drivers

Participants mentioned multiple drivers for promoting PE within research (Table 2). A key driver was that PE or the broader concept of science communication was mandated within agencies’ missions and policies.

**Table 2:** Drivers for promoting public engagement in research

Aligning public views with research agendas	Increasing the relevance of research
Benefiting patients	Inspiring children to consider science careers
Bringing together different perspectives	Legitimising research
Building in impact	Listening to the public
Delivering value for money	Maintaining a license to practice
Ensuring fair decision-making	Mandated in organisational mission
Facilitating rapid translation	Part of organisational culture
Gaining public approval	Participation is implicit in social sciences
Improving researchers’ skill-sets	Public input into the direction of science
Improving the public’s science literacy skills	Restoring the reputation of science
Improving the quality of research	Shifting research towards a problem-focus
Improving treatments	The accountability of taxpayers’ money
Increasing public support	The democratic imperative
Increasing patient choice	Transparency – keeping the public informed
Increasing public confidence in science	

Drivers were often underpinned by educational, financial, moral, philosophical, political and utilitarian rationales. For example, two colleagues from the same funding agency (one working in a PE role and the other a research funding role) talked about the multiple drivers behind promoting PE:

“*I guess there’s a lot of different drivers for doing public engagement. There’s the sort of really practical stuff, like it’s good for your communication skills, and that sort of thing, and then there’s more philosophical drivers around democratising science, and it’s the taxpayer who’s paying, and all that sort of thing. But I think the one which probably resonates most strongly with* [the funding agency] *–* [colleague’s name] *might disagree with this – is a need for us as a body to have a licence to operate. That phrase from the 2000 House of Lords report. I think they said ‘licence to practice’, actually. So if science is going to be able to move forward, then we need support, public support, and approval of what we do. And that of course means not just telling people stuff, but also giving them a voice in what we’re doing. And that public support does feed through into the ministerial, up to the ministerial level, which then, in the long term, essentially, sets our budget*”.

Different rationales for promoting PE in research were sometimes discussed in relation to specific groups. For example, accountability, democracy and transparency in science were often seen as important for public audiences, whereas improving research quality and skill development were often viewed as important for researchers, and public support and trust in science was often discussed as important for high-level decision-makers and policy actors. These differences were somewhat reflected in the perspectives of the different sub-groups of participants. Those working directly for funding agencies who discussed PE at an organisational-level tended to emphasise its importance in terms of advocacy, whereas research professionals who sat on grant review committees tended to stress the importance of PE for advancing the quality of science. In the context of the research grant process, grant review committee members hold the decision-making power and, therefore, their conception of PE for scientific excellence is perhaps most likely to be given prominence.

#### Distinctiveness

Many participants perceived the flexible and inclusive nature of PE provided funding agencies with the freedom to carve out their own distinctive PE niche and strategy. Some discussed de-prioritising certain areas of PE work to avoid duplicating the work of others. One participant, who held a PE role within their agency, spoke candidly about the importance of possessing a unique PE space within the research funding marketplace:

“*We are essentially, in many ways, in competition with each other… if we all look like we’re occupying the same space the Government asks: ‘Why do we have these different systems then?’ So there needs to be a certain element of differentiation*”.

It seems, therefore, that market forces within the research funding sector appear to incentivise distinctiveness, innovation and competition amongst funding agencies.

### Section two: PE within the research grant process

Although funding agencies seemed to be united in promoting PE within their visions and strategies, not all agencies promoted PE (or similar activities labelled as impact or communication) within the process of awarding research grants. Those who did, however, commonly took the stance that PE should be embedded within research applications generally rather than being funded separately. Only two funding agencies provided separate PE grants in addition to research grants.

#### Planning phase

Funding agencies operated managed and/or open funding streams. Whereas managed streams fund research which addresses an issue that has been strategically prioritised, open streams welcome research grant proposals that fall within the broad remit of a funding agency’s scope. Of the agencies that operated managed funding streams, only one agency that funded the most significant amount of applied health research seemed to routinely involve members of the public in discussions about research funding priorities. More commonly, funding agencies used a targeted approach, seeking public input when organisational staff and academic experts deemed it relevant to a specific topic, which was commonly the case if topics generated public controversy or involved complex ethical issues. For example, one participant, who held a funding role within their agency, stated:

“*If you think about our standard grants, or our standard fellowships, those are, I suppose, less overtly driven by public voice, per se.* […] *But there will be particular areas where there’s great sensitivity,* […] *where clearly particular engagement in the public agenda is absolutely key, to make sure that we’ve got that right. So I think at the moment, what’s fair to say is that* [seeking public opinion is] *fairly targeted, rather than blanket”.*

In setting health research agendas, PE seems more important to those funding agencies aligned to disciplines which foster research in a multiplicity of epistemological traditions rather than those which prioritise research produced in the post-positivist tradition. Rather than involving publics in all research, selective involvement in those calls or areas which have a particular ‘sensitivity’ or ethical challenge, suggests an approach to PE which is more associated with ensuring, and not disrupting, public trust in research rather than democracy and accountability, or indeed researcher development. Furthermore, these insights into how PE is used in the planning of funding calls suggest something about how PE is conceived more broadly within funding agencies. Indeed, the interview accounts suggest PE is primarily seen as a means of informing and consulting publics rather than in empowering and collaborating with them.

#### Application phase

Participants from agencies that requested information on PE or impact/communication within their grant applications commonly explained the importance of applicants providing this information in order for their application to be processed. For example, one participant who was a grant review committee member emphasised: “*It’s required and they won’t get the money unless it’s satisfactory*”. Despite this insistence, it was not always clear how PE plans featured within the research grant assessment and award process.

There was also some indication that the size of the grant made a difference to the PE expectations placed on researchers. For example, some participants discussed how, in comparison to smaller grants, large grants awarded to research centres came with greater expectations to embed PE within the institution’s research and organisational culture. Some suggested that this was because research centres had more resources and infrastructure available, and were seen as having to be more accountable to the public due to the size of their budgets.

#### Assessment phase

The process for reviewing research grant applications often followed some common steps: an initial screening phase by funding agency staff (checking for completion), external review (commonly by academics and professionals with specific expertise), and assessment by a grant review committee (primarily comprising academic members and professionals). It seemed to be rare for funding agencies to involve members of the public in the assessment process.

While funding agencies have articulated their support for PE within science, in conversations with participants about the assessment of research grants, tensions around embedding PE in the assessment process became particularly evident. For example, one participant, who held a PE role within their agency, said:

“*I think we need to probably recognise what our role is in this landscape, which is primarily about funding excellent research. We, as a* [funding agency]*, think that public engagement has an important part to play in that. And we’ve said that, quite loudly, a number of times* […] *I think that’s probably a fairly appropriate level of activity for us. Whilst personally, a lot of us involved in the field would love to make a whole lot more noise about it, I think we probably have to be a little realistic about what the role of a* [funding agency] *should be, in these things*”.

Participants commonly discussed how grants were overwhelmingly assessed on their ‘scientific excellence’, a term which was used to refer to the excellence/appropriateness of methods and research or, in the case of fellowships, to the ‘best’ scientists. Only one agency, which funded significant amounts of applied health research, indicated that PE was a key criterion for assessing research grants and an embedded part of their notion of ‘scientific excellence’. Other common factors deemed important to consider within the assessment of grants were a strategic alignment with the funders’ visions, value for money, and the research environment. Within the assessment process, PE was largely viewed as a secondary consideration and contingent factor that, in principle, may be used in the assessment process to discern between equally graded applications after the science and the other factors had been assessed.

Amongst participants there was little consensus regarding fixed criteria for good PE practice. However, it was often implied, especially by those sitting on grant review committees, that PE was judged from a utilitarian perspective of solving a scientific problem. If PE plans needed improving, this was not perceived as a problem per se as applicants were simply asked to rewrite this section as part of a conditional grant offer. Furthermore, few statements were made around what PE activities were deemed appropriate. Activities that seemed to be most commonly proposed in grant applications were forms of information provision with varying degrees of interactivity (Table 3) as opposed to exercises that provided two-way opportunities for scientists to speak with and learn from the public.

**Table 3:** Typical public engagement activities proposed within research grant applications

Advocacy organisation involvement	Lay members within steering groups
Cafe scientifique	Media work
Collaboration with the university press office	Public lectures
Delivery of workshops	School outreach work
Films	Science festival exhibitions
Interactive exhibits	Social media
Interdisciplinary conferences	Websites

Within funding agencies, perceptions of how PE contributes to good research practice seem mainly focused on informing and consulting with the public rather than creating collaborative relationships with them, prioritising this understanding of PE.

#### Monitoring phase

Not all funding agencies monitored PE, either through progress-checks or to assess effectiveness. A number of participants spoke of the challenges associated with collecting such data. Over half of the funding agencies used or intended to use a standardised research outcomes system to collect annual and end-of-grant information on research outputs. These systems focused on collecting predominantly quantitative data approximating the reach and impact of PE activities as opposed to more comprehensive information that reflected the context, procedure and details of specific activities and if and why they were deemed successful or not. Indeed, this narrow focus on procedural monitoring of PE further seems to prioritise understandings of PE as an information dissemination activity rather than a research practice that should be reported on in detail and rigorously evaluated.

## Discussion

To our knowledge, this study is the first to have focussed on conceptions of PE and how United Kingdom non-commercial agencies that fund health and medical research have interpreted and implemented their role to promote it within research. The use of a qualitative interview approach in this study provided an opportunity to go beyond published PE policies and declarations featured in funding agencies’ documentation and websites, and gain a deeper insight into how PE is actively negotiated within existing organisational systems and practices.

We anticipate that this study is likely to be of interest to an international audience due to the growing political interest in PE within research globally [4, 5]. Although the context-dependent nature of qualitative research limits the universal generalisability of the data, we believe that the findings and implications to come from our work are likely to resonate with and help inform other international contexts that have an interest in promoting PE within research.

Contrary to previous studies that suggest a common set of PE (and similar concepts such as Knowledge Exchange) definitions should be sought [17, 44], our findings indicate that the highly context-dependent nature of PE is likely to undermine and negate any attempts to form a common language and approach across funding agencies. The idiosyncratic characteristics of many funding agencies, the opportunities the different disciplines they align with afford to interact with the public, and the competitive environment in which they work, contribute to deep-rooted differences in the way PE is interpreted and operationalised across funding agencies. Our findings also indicate that the flexible nature of PE enables and legitimises funding agencies to shape the boundaries of PE within research in accordance with their own cultures, disciplines and practices. The power that funders hold within their research community indicates that these individualised working definitions are also likely to impact on research practices within those communities [33]. This creates a further challenge for researchers looking to pursue funding who require an awareness of the differing and changing conceptions of PE that agencies hold.

Previous scholars have identified numerous rationales that can drive PE within research [14, 33, 45, 46]. In the funding context, we too found this to be the case. Within most funding agencies, there appeared to be a political agenda for the promotion of PE as an advocacy tool to develop public trust and support for agencies’ respective research remits. In the research grant funding process, and particularly in the assessment phase where proposals are scrutinised largely by elected academics, there was more of a utilitarian emphasis on PE as a problem-solving tool for improving science. Indeed, we found little evidence that within research funding agencies’ practices PE was being driven by a democratic imperative, which considers the potential gains of inclusive governance and public/patient empowerment within research. While it is perhaps an intuitive choice for funders and academics to view PE as a tool to further their own agendas, it is worthwhile for those promoting and assessing PE within research and science policy to also consider the possible benefits that involving people in research can also bring to individuals and society. Developing an awareness of the underlying rationale/s by which PE is being appraised would also help in the development of evaluation work in this area, as effective indicators can only be established in light of having a clear understanding of what outcomes and possible impacts are expected to come from PE activities.

We found that, while funding agencies viewed PE promotion as a part of their role – often reinforced within organisational visions or missions – some embraced this ‘duty’ more than others. Although our study indicated that funding agencies’ relationships with the public differed, and while there are some good examples of funders facilitating and hosting collaborative PE events, it was commonplace for funding agencies to conceptualise PE as a tool to inform and consult the public as opposed to empower and collaborate with them [16]. Among the agencies that requested PE information within research grant applications, a broad range of public-facing activities was accepted as PE, many of which were manifestations of information provision. Commonly cited consultation activities were advocacy organisation involvement and lay members within steering groups. These types of public consultation are perhaps easier to incorporate into research than other forms of PE [16], and may be viable options for many researchers wanting to involve the public in research. However, truly participatory forms of PE appear much harder to achieve [47].

This study also highlighted the tension that exists between embedding PE within the research grant process and traditional conceptions of ‘scientific excellence’. The increased political pressure within health and medicine to demonstrate the societal and economic impacts of research has popularised the concept of socially embedded research and practices such as public dialogue and participation [27, 48, 49]. However, United Kingdom funders are also under considerable pressure to produce the ‘best’ scientists and the ‘best’ research within the international arena, which we found often appeared to relate to technical endeavour and ‘objective science’, and seemed to conflict with the subjective nature of PE.

Regardless of the pressures to involve the public in research, we found funders prioritised traditional notions of ‘scientific excellence’ within the shaping and assessment of research. PE rarely formed a key assessment criterion within research grant decisions. Only one agency, which funded significant amounts of applied health research, included participatory notions of PE as a key criterion for assessing research grants and as an aspect of ‘scientific excellence’. As applied health research often has a closer relationship to the public and patients than some types of basic health and medical sciences, there is potentially a greater opportunity and a clearer pathway for involving individuals within the development and execution of this particular kind of research. However, given the imperative for PE transcends disciplinary boundaries, perhaps all kinds of research associated with health and medicine should re-evaluate their conception of scientific excellence to include the notion of societal impact in a meaningful way.

Similar to previous research, we found that funding agencies’ explicit PE policies rarely seemed to materialise in any serious sense into the operational business of funding research [26, 31, 32]. It has been previously suggested that the current system of research grant assessment is not well suited to appraising socially embedded research [50]. However, some also suggest that shifting away from the current assessment system and towards a more pragmatic market logic approach would likely damage the integrity of science [51]. Our study raises an important question: how do research funding agencies find the appropriate balance between assessing technical rigour, demonstrated through traditional conceptions of scientific excellence, and social robustness, achieved through engaging citizens in research? Although we anticipate that this balance is likely to differ across research disciplines and subjects, it is important, even for the most basic ‘blue skies’ research, to consider the potential value that activities such as PE can bring to the different stakeholders involved, as much non-commercial health and medical research is funded by and in the interest of the public. Indeed, PE has the potential to enhance the ecological validity of research studies, which can bring gains to funders, researchers and the public alike [1, 2].

Although we have identified some novel findings that help to understand and explain some of the PE policies and practices found to exist within United Kingdom non-commercial agencies that fund health and medical research, we do not wish to claim our findings are exhaustive. Not only is the nature of PE and its relationship with science complex, but so too is the non-commercial research sector and the numerous actors and influences that operate within it. Our study focussed on how PE is interpreted by funding agencies and how it featured within the research grant process, but in addition to this, it is important to recognise that most funding agencies also promote PE through a number of other avenues such as by working closely with and investing resources in the university sector to create a culture change, commissioning PE activities and research, and supporting researchers who want to carry out PE.

It is important to acknowledge that participants’ accounts within our study may not always portray an accurate representation of their funding agency’s policies and practices. In order to respond to this potential limitation and reduce inaccuracy within the data, we aimed to interview three individuals affiliated to each participating funding agency, alongside using data from their websites. Additionally, during interviews we clearly stated our interest in agency policies and practices as opposed to personal views. The area of PE is particularly dynamic and PE policies and practices are likely to change and develop from year-to-year. This research then, although bounded by contemporary policies and practices of specific agencies, aims to offer more broad insights into the context-specific nature of this dynamic area.

## Conclusions

Our study has highlighted the role of funding agencies in shaping how PE is defined and interpreted within health and medical research communities. In particular, our analysis suggests that agencies working within specific areas of health and medicine can promote particular definitions of PE and aligned practices which determine the boundaries in which researchers working in these specific areas understand and practice PE. Indeed, the profound differences in the way PE is interpreted and operationalised across funding agencies, and agencies’ interests in maintaining a distinctive space in the PE landscape, create challenges for those aiming to unify conceptions of PE and indeed researchers aiming to include appropriate PE plans in funding applications.

Furthermore, our study has highlighted how the research grant process works to privilege some groups’ conceptions of PE and its purpose. Some agencies seem to value PE as a means of informing and consulting the public but not necessarily collaborating with or empowering them. During the assessment phase of the research funding process, the impact of prioritising specific drivers is evident. Academic reviewers’ are afforded a key role in promoting PE as a means of supporting scientific excellence. These limited conceptions of PE, at key stages in the funding process, do not represent the more egalitarian basis for engagement and involvement of the public as a key means of democratising health research.
